# Letter to the editor regarding ‘Is combined antidepressant medication (ADM) and psychotherapy better than either monotherapy at preventing suicide attempts and other psychiatric serious adverse events for depressed patients? A rare events meta-analysis’

**DOI:** 10.1017/S0033291724002071

**Published:** 2024-09

**Authors:** Alexander Lisinski, Fredrik Hieronymus

**Affiliations:** 1 Department of Pharmacology, Institute of Neuroscience and Physiology, Sahlgrenska Academy, University of Gothenburg, Gothenburg, Sweden; 2 Department of Psychiatry, Sahlgrenska University Hospital, Region Västra Götaland, Gothenburg, Sweden

**Keywords:** antidepressants, combined treatment, depression, major depressive disorder, meta-analysis, pharmacology, psychiatry, psychoterapy

## Letter

A recent meta-analysis published in this journal (Zainal, 2024) found combined treatment with antidepressants and psychotherapy (ADM-PSY) to be inferior to psychotherapy alone (PSY), in preventing a combined outcome of suicide attempts, psychiatric emergency department visits, psychiatric hospitalization, and/or suicide death (Peto odds-ratio 1.96 [1.20–3.20]).

This finding is important considering the much-discussed possibility of antidepressants having suicidogenic effects (Healy & Whitaker, 2003; Stone et al., 2009). It is also surprising since, except for those aged 24 years or below, analyses of the much larger population of placebo-controlled trials have not found any indications that antidepressants have pro-suicidal effects (Stone et al., 2009). A significant difference in a much smaller population where all participants received psychotherapy – ostensibly reducing suicidality – is thus both unexpected and interesting. However, scrutinizing the data for the ADM-PSY to PSY comparison (Fig. 2 in Zainal, 2024) suggests that the significant difference in favor of PSY can be explained by errors in data extraction and synthesis.

The three studies with the most weight (March 2004, March 2007 and Vitiello 2009, combined weight 52.7%) in the meta-analysis are all from the same trial: The NIMH-funded Treatment of Adolescents with Depression Study (TADS). The same patients are thus included three times. Two of these inclusions (March 2004 and March 2007) are erroneous also in the sense that the reported figures include suicidal ideation (Vitiello et al., 2009), which does not form part of the combined outcome that the author reports to be studying.

For the fourth most influential study (Browne 2002, weight 11.4%), the author states that the rates of psychiatric hospitalization were 0% for interpersonal psychotherapy alone and 4.3% for interpersonal psychotherapy combined with sertraline (see Table 1; Zainal, 2024). This is also incorrect since these figures correspond to all serious adverse events (SAEs). Notably, this was not a study of patients with major depressive disorder, as stated in Table 1 in the article, but of patients with dysthymic disorder.

Continuing to the fifth most influential study (Davey 2019, weight 8.5%) the author here unfortunately appears to have switched groups; there were thus not 5 events (suicide attempts) in the combined therapy arm and 1 in the psychotherapy alone arm, but the other way around (n.b. the author reports the events correctly in Table 1).

More errors and inconsistencies were found, e.g., the study by Huijbers and colleagues (weight 5.6%) did not include a PSY-only group (Huijbers et al., 2015). There is, however, another – larger – publication by Huijbers in which patients who preferred psychotherapy were withdrawn from ADM (Huijbers et al., 2016). Since the author included other partial withdrawal studies, perhaps this was the one intended to be cited? We were unable to find enough data in the larger Huijbers publication to allow for inclusion. Similarly, the study by Vitriol and colleagues did not compare PSY-only to ADM-PSY, but to *standard treatment*, with most participants in both groups receiving pharmacotherapy. Finally, one suicidal event in the study by Hollon and colleagues seems to have been incorrectly assigned to ADM-PSY instead of to ADM-only.

**Figure 1.**
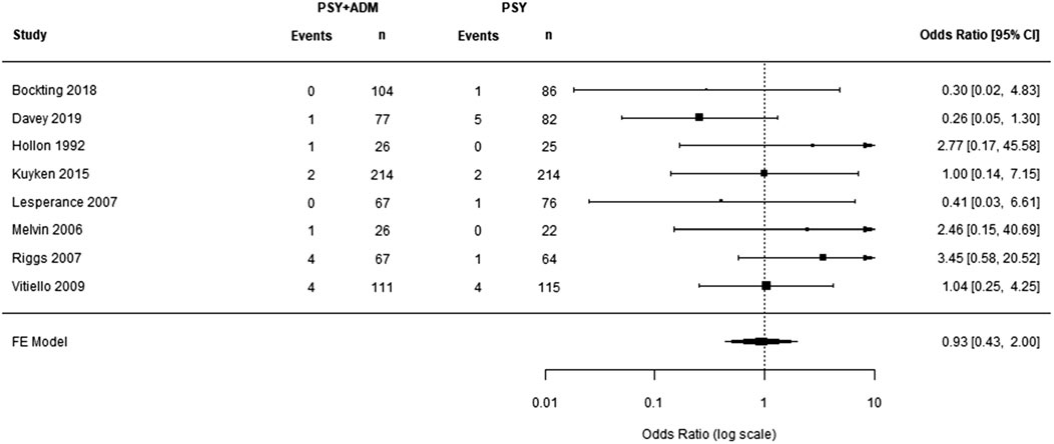
Forest plot for treatment effect comparing combined treatment with antidepressants and placebo to psychotherapy-only, using corrected numbers of events. Events refer to the number of patient(s) within a specific treatment arm that had a negative treatment outcome (i.e. suicide attempt, psychiatric emergency department visit, psychiatric hospitalization, and/or suicide death). ADM, antidepressant medication; CI, confidence interval; FE, fixed effects; PSY, psychotherapy.

Correcting the errors identified, the number of included events dropped from 47 and 22 for ADM-PSY and PSY-only, respectively, to 13 and 14. We were unable to fully replicate the analysis by Zainal since the *rmeta* package – as far as we can tell – does not have a built-in Peto method. Using the rma.peto method in the *metafor package,* we could replicate the mean effect reported, albeit with a slightly different CI (1.96, 1.22–3.15). Using this method to analyze the corrected data, the pooled OR was 0.93 (0.43–2.00; Fig. 1).

We do not intend for this to be a full replication (or rebuttal) of the original analyses. However, data extraction errors were not exclusive to the analysis contrasting ADM-PSY to PSY-only. For example, in the comparison of ADM-only to PSY-only, the TADS study was again included three times. This was the case also for the analysis comparing ADM-PSY to ADM-only, where additionally the Adolescent Depression Antidepressant and Psychotherapy Trial (ADAPT) was included twice (Goodyer 2007 and Wilkinson 2011). In the same analysis, the author seems to have missed to include events from one of the combined treatment arms in the study by Brent and colleagues (weight 13.7%) leading to an underestimation of 10 events in the combined group. For the study by Michel and co-workers (weight 12.6%) we could not identify the number of patients extracted in the original publication, nor the number of events. The study by Kocsis does not seem to have included psychotherapy, only comparing antidepressants to placebo, and in the study by Wei, 77 out of 82 participants assigned to cognitive therapy did not receive any therapy, the only other active intervention being performed by telephone, for another 60 participants.

As noted also by Zainal in the discussion, it is very difficult to get accurate estimates of combined endpoints of harm since trials in general – and older trials in particular – do not provide thorough AE reporting (Hieronymus, Lisinski, Näslund, & Eriksson, 2018). This is true also for psychotherapy trials (Klatte, Strauss, Fluckiger, & Rosendahl, 2023). While suicides and suicide attempts were often detailed with some regularity in the original articles, useable data on the two other endpoints included by the author (psychiatric hospitalizations and psychiatric emergency department visits) were rarely present. The use of a combined endpoint for which full data is almost never available adds a veneer of comprehensiveness that the available literature unfortunately cannot provide.

While these are unfortunate drawbacks of the literature that are likely impossible to compensate for, the study by Zainal et al. also seems to include many issues that could have been avoided. We have identified several inaccuracies which, taken together, significantly affect the reported results and conclusions. As stated, it is not our intention to do a full re-analysis of the data – nor to conduct our own literature search – but given the scope of the errors we believe that such an effort is motivated and that the paper need to be corrected so as to properly reflect the available evidence.
